# Supplementary figures and images for: Effective Prophylaxis of COVID-19 in Rhesus Macaques Using a Combination of Two Parenterally-Administered SARS-CoV-2 Neutralizing Antibodies
Source: Front Cell Infect Microbiol. 2021 Nov 18;11:753444. doi: 10.3389/fcimb.2021.753444 (PMC8637877; doi:10.3389/fcimb.2021.753444)

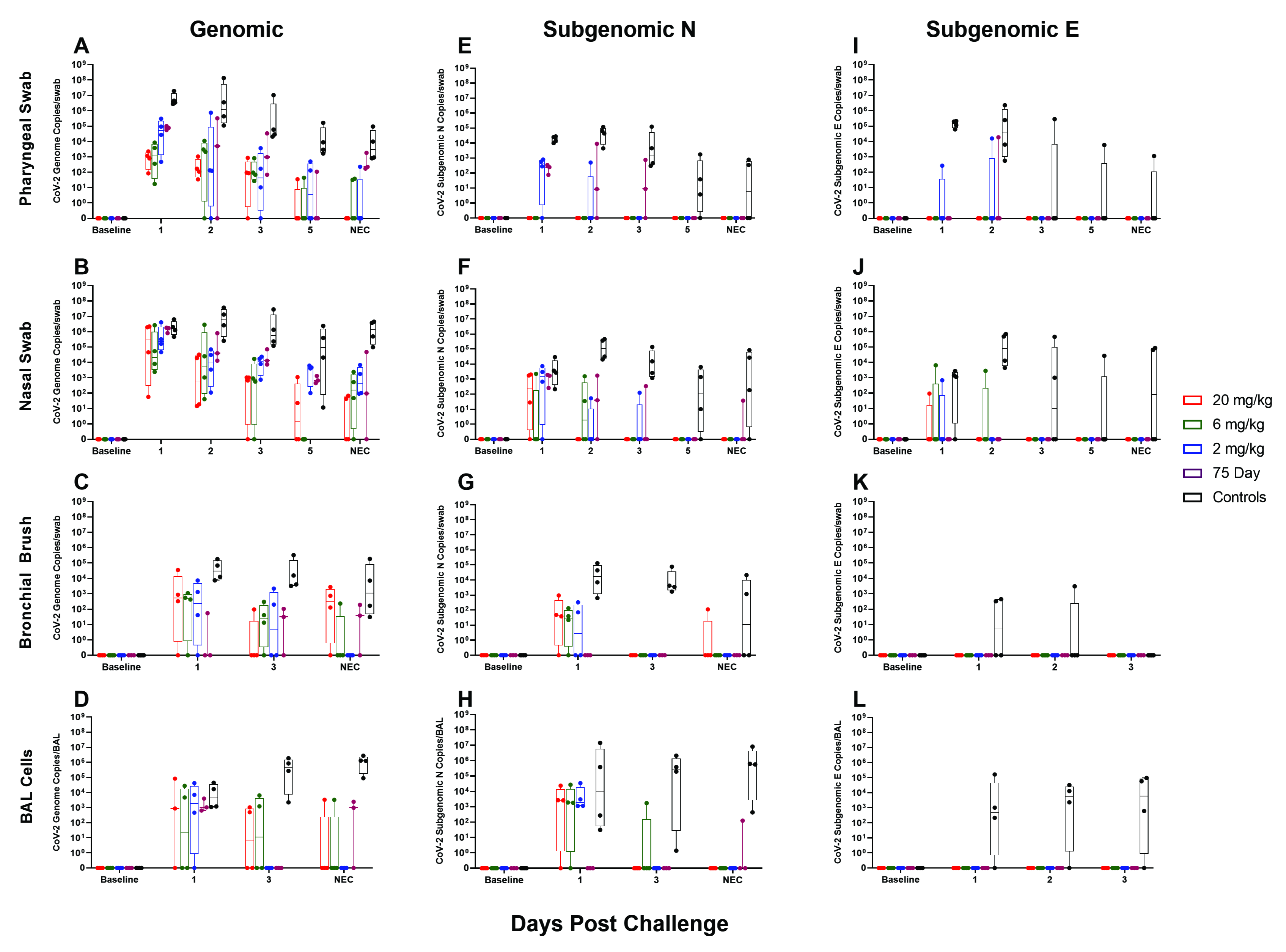

Supplement: Supplementary file 1 [file Image_1.tif]

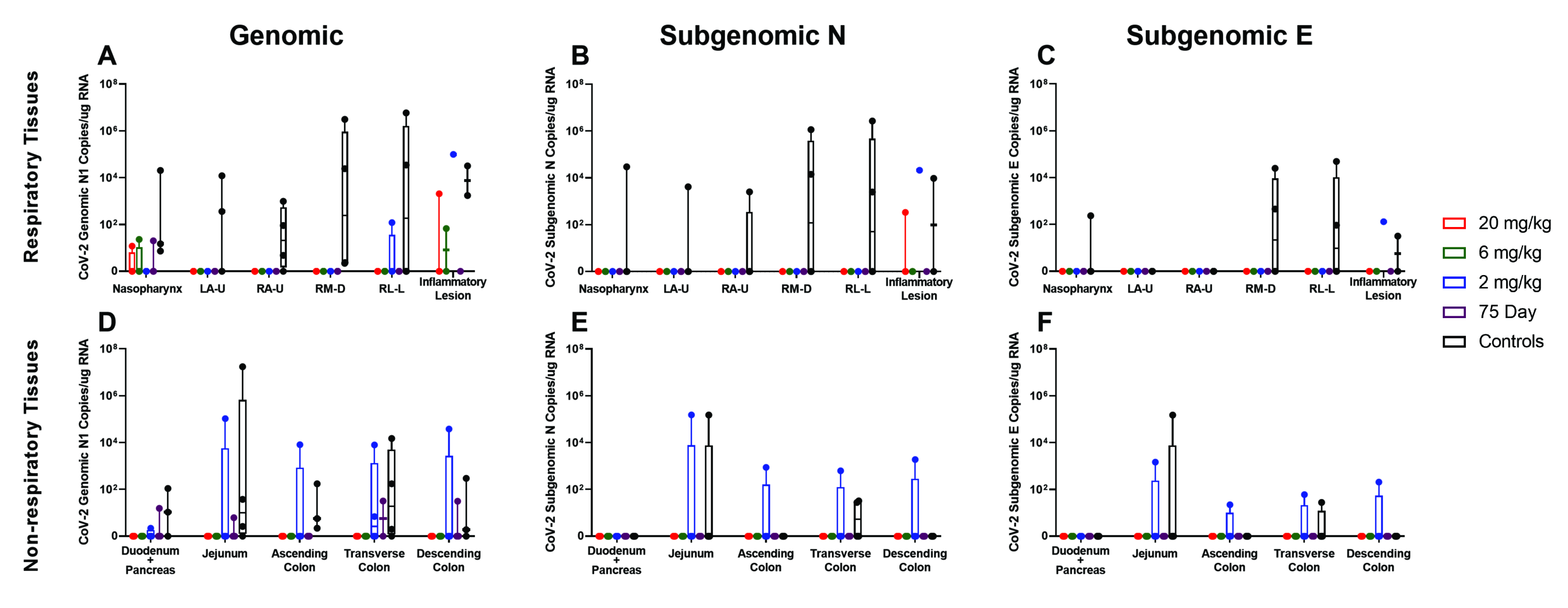

Supplement: Supplementary file 2 [file Image_2.tif]

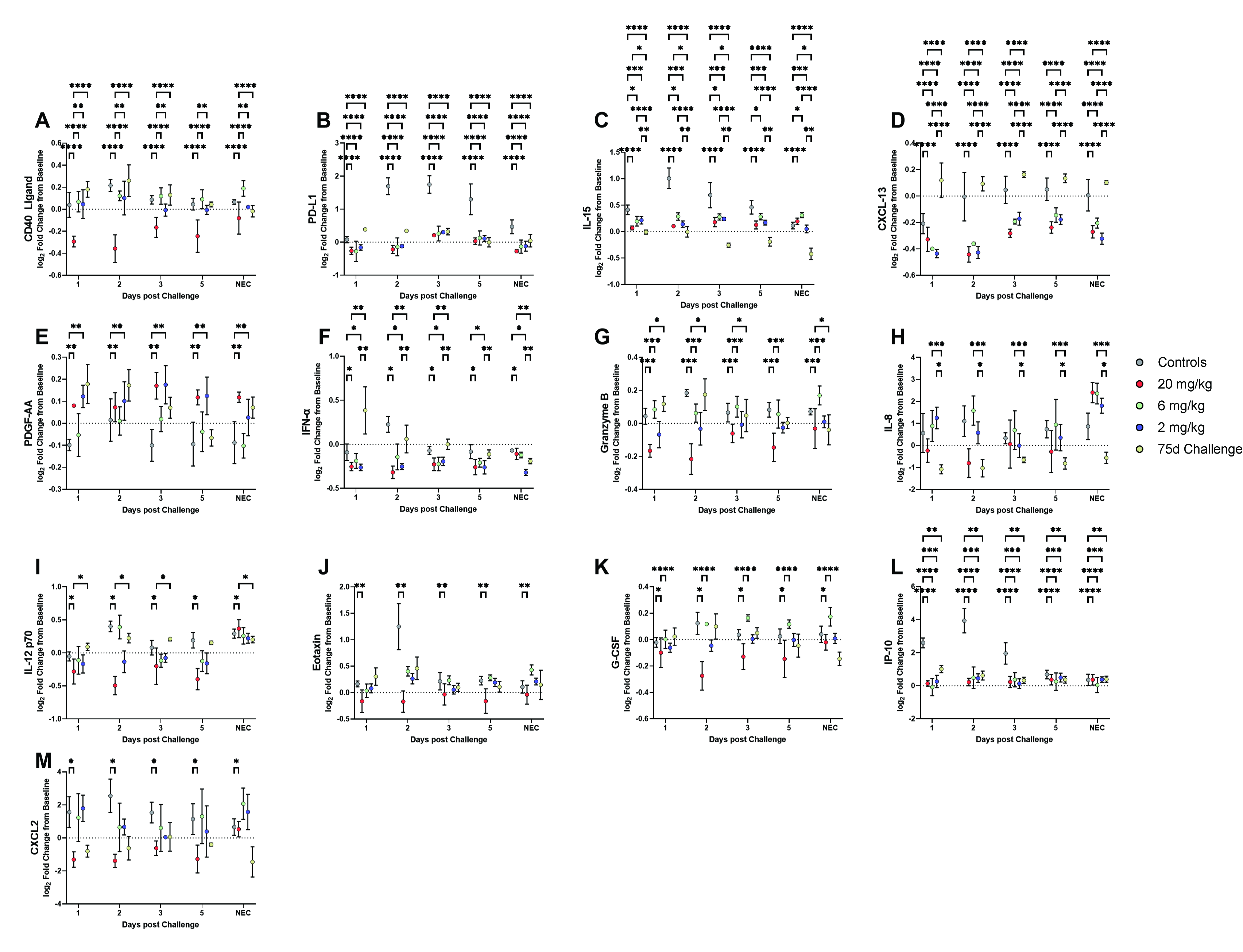

Supplement: Supplementary file 3 [file Image_3.tif]

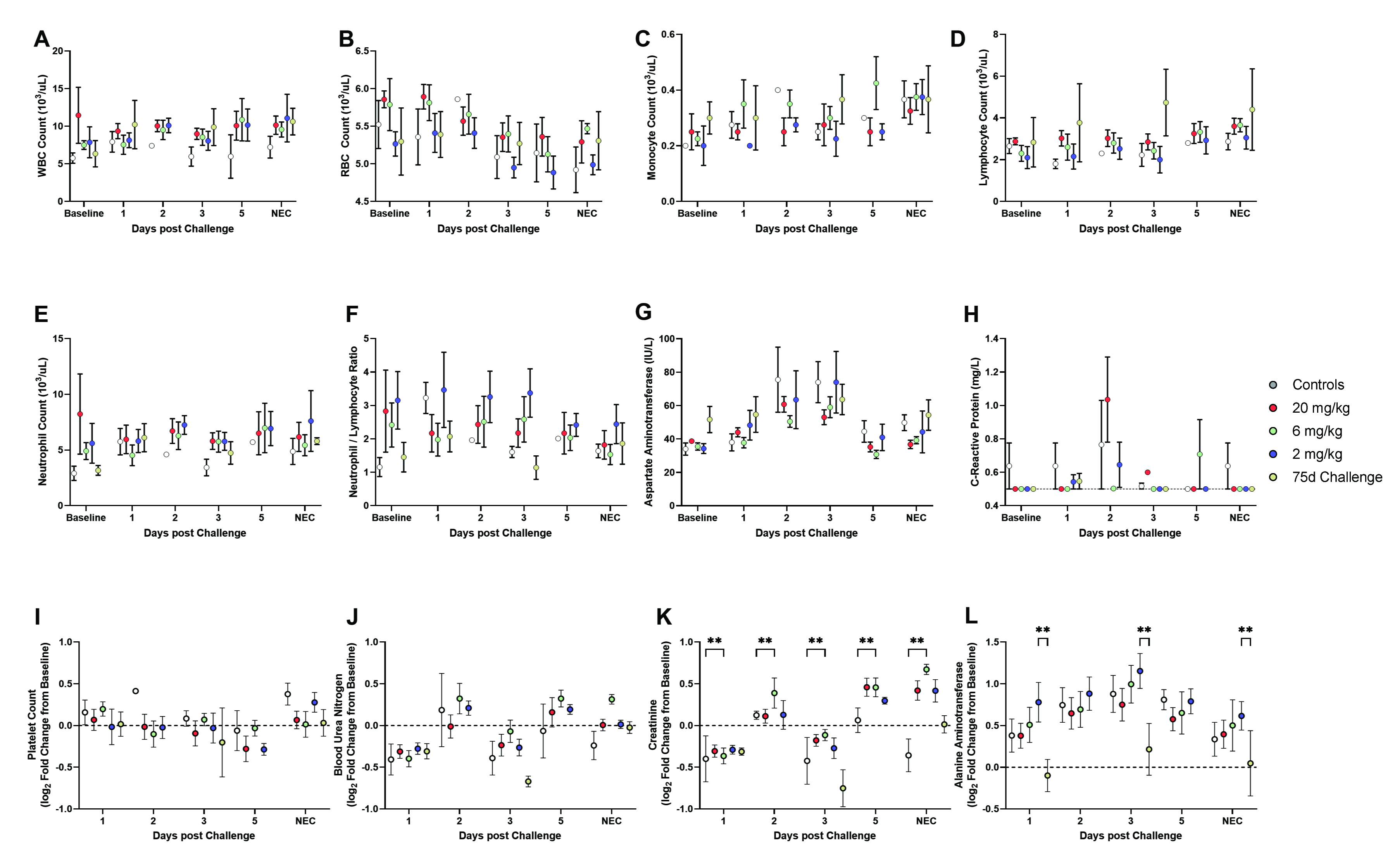

Supplement: Supplementary file 4 [file Image_4.tif]
