# Supplementary material for: Effective Prophylaxis of COVID-19 in Rhesus Macaques Using a Combination of Two Parenterally-Administered SARS-CoV-2 Neutralizing Antibodies
Source: Front Cell Infect Microbiol. 2021 Nov 18;11:753444. doi: 10.3389/fcimb.2021.753444 (PMC8637877; doi:10.3389/fcimb.2021.753444)
Supplement: Supplementary file 5 [file Table_1.pdf]

Animal	Species	Age (years)	Source	Sex	Weight (kg)	Viral Dose ($TCID_{50}$)	Exposure Route	mAb Dose (mg/kg)	Challenged post Infusion (Days)
**LM74**	*Macaca mulatta*	4	TNPRC	Male	6.2	2.0×10^6	IT/IN	0	3
**IK92**	*Macaca mulatta*	11	TNPRC	Male	6.9	2.0×10^6	IT/IN	0	3
**KF89**	*Macaca mulatta*	8	TNPRC	Male	8.2	2.0×10^6	IT/IN	0	3
**LM30**	*Macaca mulatta*	4	TNPRC	Male	8.1	2.0×10^6	IT/IN	0	3
**LN97**	*Macaca mulatta*	4	TNPRC	Male	4.3	2.0×10^6	IT/IN	20	3
**LR09**	*Macaca mulatta*	4	TNPRC	Male	4.8	2.0×10^6	IT/IN	20	3
**MD42**	*Macaca mulatta*	3	TNPRC	Male	5.0	2.0×10^6	IT/IN	20	3
**MF22**	*Macaca mulatta*	3	TNPRC	Male	5.0	2.0×10^6	IT/IN	20	3
**MC12**	*Macaca mulatta*	3	TNPRC	Male	5.3	2.0×10^6	IT/IN	6	3
**LR41**	*Macaca mulatta*	4	TNPRC	Male	5.7	2.0×10^6	IT/IN	6	3
**ME55**	*Macaca mulatta*	3	TNPRC	Male	5.8	2.0×10^6	IT/IN	6	3
**LV40**	*Macaca mulatta*	4	TNPRC	Male	5.6	2.0×10^6	IT/IN	6	3
**LM12**	*Macaca mulatta*	4	TNPRC	Male	6.1	2.0×10^6	IT/IN	2	3
**LT54**	*Macaca mulatta*	3	TNPRC	Male	6.2	2.0×10^6	IT/IN	2	3
**MG10**	*Macaca mulatta*	3	TNPRC	Male	6.0	2.0×10^6	IT/IN	2	3
**IR17**	*Macaca mulatta*	11	TNPRC	Male	6.3	2.0×10^6	IT/IN	2	3
**LP79**	*Macaca mulatta*	4	TNPRC	Male	5.5	2.0×10^6	IT/IN	20	75
**LR93**	*Macaca mulatta*	4	TNPRC	Male	6.0	2.0×10^6	IT/IN	20	75
**MC61**	*Macaca mulatta*	3	TNPRC	Male	5.7	2.0×10^6	IT/IN	20	75
