# Supplementary material for: Effective Prophylaxis of COVID-19 in Rhesus Macaques Using a Combination of Two Parenterally-Administered SARS-CoV-2 Neutralizing Antibodies
Source: Front Cell Infect Microbiol. 2021 Nov 18;11:753444. doi: 10.3389/fcimb.2021.753444 (PMC8637877; doi:10.3389/fcimb.2021.753444)
Supplement: Supplementary file 6 [file Table_2.pdf]

Target	Primer/Probe Designation	Sequence
Genomic Nucleocapsid	2019-nCoV_N1-F	5'-GAC CCC AAA ATC AGC GAA AT-3'
	2019-nCoV_N1-R	5'-TCT GGT TAC TGC CAG TTG AAT CTG-3'
	2019-nCoV_N1-P	5'-FAM-ACC CCG CAT TAC GTT TGG TGG ACC-BHQ-3'
Subgenomic Envelope	SgE-F	5'-CGA TCT CTT GTA GAT CTG TTC TC-3'
	SgE-R	5'-T GTG TGC GTA CTG CTG CAA TAT-3'
	SgE-P	5'-FAM-ACA CTA GCC ATC CTT ACT GCG CTT CG-BHQ-3'
Subgenomic Nucleocapsid	SgN-F	5'-CGA TCT CTT GTA GAT CTG TTC TC-3'
	SgN-R	5'-GGT GAA CCA AGA CGC AGT AT-3'
	SgN-P	5'-56-FAM/TAA CCA GAA/ZEN/TGG AGA ACG CAG TGG G/3IABkFQ/-3'
