# Supplementary material for: Effective Prophylaxis of COVID-19 in Rhesus Macaques Using a Combination of Two Parenterally-Administered SARS-CoV-2 Neutralizing Antibodies
Source: Front Cell Infect Microbiol. 2021 Nov 18;11:753444. doi: 10.3389/fcimb.2021.753444 (PMC8637877; doi:10.3389/fcimb.2021.753444)
Supplement: Supplementary file 7 [file Table_3.pdf]

Genomic			p-value	Symbol
	Pharyngeal			
		20 mg/kg vs. control	0.0043	**
		6 mg/kg vs. control	0.0154	*
		2 mg/kg vs. control	0.2738	ns
		Delayed Challenge	0.9173	ns
	Nasal			
		20 mg/kg vs. control	0.1307	ns
		6 mg/kg vs. control	0.0804	ns
		2 mg/kg vs. control	0.2738	ns
		Delayed Challenge	>0.9999	ns
	Bronchial Brush			
		20 mg/kg vs. control	0.6669	ns
		6 mg/kg vs. control	0.0801	ns
		2 mg/kg vs. control	0.1205	ns
		Delayed Challenge	0.0087	**
	BAL Cells			
		20 mg/kg vs. control	0.0677	ns
		6 mg/kg vs. control	0.1409	ns
		2 mg/kg vs. control	0.0621	ns
		Delayed Challenge	0.1252	ns

Subgenomic N			p-value	Symbol
	Pharyngeal			
		20 mg/kg vs. control	0.0036	**
		6 mg/kg vs. control	0.0036	**
		2 mg/kg vs. control	0.3367	ns
		Delayed Challenge	0.7044	ns
	Nasal			
		20 mg/kg vs. control	0.0272	*
		6 mg/kg vs. control	0.0101	*
		2 mg/kg vs. control	0.2719	ns
		Delayed Challenge	0.3218	ns
	Bronchial Brush			
		20 mg/kg vs. control	0.2849	ns
		6 mg/kg vs. control	0.0685	ns
		2 mg/kg vs. control	0.0685	ns
		Delayed Challenge	0.0051	**
	BAL Cells			
		20 mg/kg vs. control	0.7055	ns
		6 mg/kg vs. control	0.8805	ns
		2 mg/kg vs. control	>0.9999	ns
		Delayed Challenge	0.0163	*

Subgenomic E			p-value	Symbol
	Pharyngeal			
		20 mg/kg vs. control	0.0056	**
		6 mg/kg vs. control	0.0056	**
		2 mg/kg vs. control	0.1177	ns
		Delayed Challenge	0.1125	ns
	Nasal			
		20 mg/kg vs. control	0.0259	*
		6 mg/kg vs. control	0.0399	*
		2 mg/kg vs. control	0.0322	*
		Delayed Challenge	0.0124	*
	Bronchial Brush			
		20 mg/kg vs. control	0.1006	ns
		6 mg/kg vs. control	0.1006	ns
		2 mg/kg vs. control	0.1006	ns
		Delayed Challenge	0.1527	ns
	BAL Cells			
		20 mg/kg vs. control	0.0033	**
		6 mg/kg vs. control	0.0033	**
		2 mg/kg vs. control	0.0033	**
		Delayed Challenge	0.0078	**
