# Supplementary material for: Effective Prophylaxis of COVID-19 in Rhesus Macaques Using a Combination of Two Parenterally-Administered SARS-CoV-2 Neutralizing Antibodies
Source: Front Cell Infect Microbiol. 2021 Nov 18;11:753444. doi: 10.3389/fcimb.2021.753444 (PMC8637877; doi:10.3389/fcimb.2021.753444)
Supplement: Supplementary file 8 [file Table_4.pdf]

Genomic

Pharyngeal		p-value	Symbol
	2 mg/kg vs. control day 1	0.0286	*
	2 mg/kg vs. control day 2	0.1143	ns
	2 mg/kg vs. control day 3	0.0286	*
	2 mg/kg vs. control day 5	0.0286	*
	2 mg/kg vs. control NEC	0.0286	*
	Delayed Challenge vs. control day 1	0.0571	ns
	Delayed Challenge vs. control day 2	0.1143	ns
	Delayed Challenge vs. control day 3	0.2286	ns
	Delayed Challenge vs. control day 5	0.0571	ns
	Delayed Challenge vs. control NEC	0.2286	ns
Nasal			
	20 mg/kg vs. control day 1	0.4857	ns
	20 mg/kg vs. control day 2	0.0286	*
	20 mg/kg vs. control day 3	0.0286	*
	20 mg/kg vs. control day 5	0.1143	ns
	20 mg/kg vs. control NEC	0.0286	*
	6 mg/kg vs. control day 1	0.2	ns
	6 mg/kg vs. control day 2	0.1143	ns
	6 mg/kg vs. control day 3	0.2286	ns
	6 mg/kg vs. control day 5	0.0286	*
	6 mg/kg vs. control NEC	0.2286	ns
	2 mg/kg vs. control day 1	0.2	ns
	2 mg/kg vs. control day 2	0.0286	*
	2 mg/kg vs. control day 3	0.0286	*
	2 mg/kg vs. control day 5	0.3429	ns
	2 mg/kg vs. control NEC	0.0286	*
	Delayed Challenge vs. control day 1	0.8571	ns
	Delayed Challenge vs. control day 2	0.1143	ns
	Delayed Challenge vs. control day 3	0.0571	ns
	Delayed Challenge vs. control day 5	0.4	ns
	Delayed Challenge vs. control NEC	0.0571	ns
Bronchial Brush			
	20 mg/kg vs. control day 1	0.1143	ns
	20 mg/kg vs. control day 3	0.0286	*
	20 mg/kg vs. control NEC	0.4857	ns
	6 mg/kg vs. control day 1	0.0286	*
	6 mg/kg vs. control day 3	0.0286	*
	6 mg/kg vs. control NEC	0.0857	ns
	2 mg/kg vs. control day 1	0.0286	*
	2 mg/kg vs. control day 3	0.0286	*
	2 mg/kg vs. control NEC	0.0286	*
BAL Cells			
	20 mg/kg vs. control day 1	0.6286	ns
	20 mg/kg vs. control day 3	0.0286	*
	20 mg/kg vs. control NEC	0.0286	*
	6 mg/kg vs. control day 1	0.4286	ns
	6 mg/kg vs. control day 3	0.0571	ns
	6 mg/kg vs. control NEC	0.0286	*
	2 mg/kg vs. control day 1	0.4857	ns
	2 mg/kg vs. control day 3	0.0286	*
	2 mg/kg vs. control NEC	0.0286	*
	Delayed Challenge vs. control day 1	0.2286	ns
	Delayed Challenge vs. control day 3	0.0571	ns
	Delayed Challenge vs. control NEC	0.0571	ns

Subgenomic N

Pharyngeal		p-value	Symbol
	2 mg/kg vs. control day 1	0.0286	*
	2 mg/kg vs. control day 2	0.0286	*
	2 mg/kg vs. control day 3	0.0286	*
	2 mg/kg vs. control day 5	0.1429	ns
	2 mg/kg vs. control NEC	0.4286	ns
	Delayed Challenge vs. control day 1	0.0571	ns
	Delayed Challenge vs. control day 2	0.1143	ns
	Delayed Challenge vs. control day 3	0.2286	ns
	Delayed Challenge vs. control day 5	0.4	ns
	Delayed Challenge vs. control NEC	>0.9999	ns
Nasal			
	2 mg/kg vs. control day 1	0.6857	ns
	2 mg/kg vs. control day 2	0.0286	*
	2 mg/kg vs. control day 3	0.0286	*
	2 mg/kg vs. control day 5	0.1429	ns
	2 mg/kg vs. control NEC	0.1429	ns
	Delayed Challenge vs. control day 1	0.4	ns
	Delayed Challenge vs. control day 2	0.0571	ns
	Delayed Challenge vs. control day 3	0.0571	ns
	Delayed Challenge vs. control day 5	0.1429	ns
	Delayed Challenge vs. control NEC	0.2	ns
Bronchial Brush			
	20 mg/kg vs. control day 1	0.0571	ns
	20 mg/kg vs. control day 3	0.0286	*
	20 mg/kg vs. control NEC	0.4286	ns
	6 mg/kg vs. control day 1	0.0286	*
	6 mg/kg vs. control day 3	0.0286	*
	6 mg/kg vs. control NEC	0.4286	ns
	2 mg/kg vs. control day 1	0.0286	*
	2 mg/kg vs. control day 3	0.0286	*
	2 mg/kg vs. control NEC	0.4286	ns
BAL Cells			
	20 mg/kg vs. control day 1	0.6857	ns
	20 mg/kg vs. control day 3	0.0286	*
	20 mg/kg vs. control NEC	0.0286	*
	6 mg/kg vs. control day 1	0.6857	ns
	6 mg/kg vs. control day 3	0.0571	ns
	6 mg/kg vs. control NEC	0.0286	*
	2 mg/kg vs. control day 1	>0.9999	ns
	2 mg/kg vs. control day 3	0.0286	*
	2 mg/kg vs. control NEC	0.0286	*

Subgenomic E

Pharyngeal		p-value	Symbol
	2 mg/kg vs. control day 1	0.0286	*
	2 mg/kg vs. control day 2	0.0857	ns
	2 mg/kg vs. control day 3	>0.9999	ns
	2 mg/kg vs. control day 5	>0.9999	ns
	2 mg/kg vs. control NEC	>0.9999	ns
	Delayed Challenge vs. control day 1	0.0571	ns
	Delayed Challenge vs. control day 2	0.2	ns
	Delayed Challenge vs. control day 3	>0.9999	ns
	Delayed Challenge vs. control day 5	>0.9999	ns
	Delayed Challenge vs. control NEC	>0.9999	ns
Bronchial Brush			
	20 mg/kg vs. control day 1	0.4286	ns
	20 mg/kg vs. control day 3	>0.9999	ns
	20 mg/kg vs. control NEC	>0.9999	ns
	6 mg/kg vs. control day 1	0.4286	ns
	6 mg/kg vs. control day 3	>0.9999	ns
	6 mg/kg vs. control NEC	>0.9999	ns
	2 mg/kg vs. control day 1	0.4286	ns
	2 mg/kg vs. control day 3	>0.9999	ns
	2 mg/kg vs. control NEC	>0.9999	ns
	Delayed Challenge vs. control day 1	0.4286	ns
	Delayed Challenge vs. control day 3	>0.9999	ns
	Delayed Challenge vs. control NEC	>0.9999	ns
