# Supplementary material for: Effective Prophylaxis of COVID-19 in Rhesus Macaques Using a Combination of Two Parenterally-Administered SARS-CoV-2 Neutralizing Antibodies
Source: Front Cell Infect Microbiol. 2021 Nov 18;11:753444. doi: 10.3389/fcimb.2021.753444 (PMC8637877; doi:10.3389/fcimb.2021.753444)
Supplement: Supplementary file 9 [file Table_5.pdf]

	20 mg/kg challenged				6 mg/kg challenged				2 mg/kg challenged				mAb delayed challenge			mAb CTRLs		Naive	
Microscopic Findings	LN97	LR09	MD42	MF22	LR41	LV40	MC12	ME55	IR17	LM12	LT54	MG10	LP79	LR93	MC61	IK92	LM74	LM30	KF89
Interstitial inflammation	++	++	+	+	+	++	++	++	++	++	++	++	-	+	+	+++	++	++	++++
Type II pneumocyte hyperplasia	-	++	+	-	-	-	++	++	-	-	-	-	-	-	-	+++	-	++	+++
Hyaline membrane formation	-	-	-	-	-	-	-	+	-	-	-	-	-	-	-	+	-	-	-
BALT hyperplasia	-	-	+	-	-	-	-	-	-	-	-	-	+	+	+	-	-	+	-
Pharyngitis	+++	-	-	-	-	++	-	++	-	+++	-	-	-	-	-	+	-	-	-
Nephritis	++	-	++	+++	+	-	-	+	-	+	-	-	-	-	-	-	-	-	-
Gastrointestinal inflammation	+++	++	++	+++	-	+++	++	+++	++	+++	+++	++	-	-	-	++	++	+++	++++
Myocarditis	-	-	+	-	-	+	-	-	-	++	-	++	-	-	-	-	-	-	-
Thrombosis	-	-	-	-	+	-	-	-	-	-	-	-	-	-	-	-	-	-	-
Bronchial lymph node hyperplasia	-	-	++	++	+++	-	+++	+++	-	-	+++	++	-	-	-	++	+++	-	-
Mesenteric lymph node hyperplasia	++	-	-	-	-	-	-	-	-	-	-	-	++	++	++	-	-	-	-
Splenic lymphoid hyperplasia	++	-	++++	-	++	-	++	+++	-	-	-	++	-	++	+++	++	-	++	-
